# Modeling acute care utilization: practical implications for insomnia patients

**DOI:** 10.1038/s41598-023-29366-6

**Published:** 2023-02-07

**Authors:** Farid Chekani, Zitong Zhu, Rezaul Karim Khandker, Jizhou Ai, Weilin Meng, Emma Holler, Paul Dexter, Malaz Boustani, Zina Ben Miled

**Affiliations:** 1 Merck & Co., Inc., Rahway, NJ 07065 USA; 2 Computer Science, IUPUI, Indianapolis, IN 46202 USA; 3 School of Public Health, Indiana University, Bloomington, IN 47405 USA; 4 School of Medicine, Indiana University, Indianapolis, IN 46202 USA; 5 Regenstrief Institute, Indianapolis, IN 46202 USA; 6 Electrical and Computer Engineering, IUPUI, Indianapolis, IN 46202 USA

**Keywords:** Health care economics, Machine learning

## Abstract

Machine learning models can help improve health care services. However, they need to be practical to gain wide-adoption. In this study, we investigate the practical utility of different data modalities and cohort segmentation strategies when designing models for emergency department (ED) and inpatient hospital (IH) visits. The data modalities include socio-demographics, diagnosis and medications. Segmentation compares a cohort of insomnia patients to a cohort of general non-insomnia patients under varying age and disease severity criteria. Transfer testing between the two cohorts is introduced to demonstrate that an insomnia-specific model is not necessary when predicting future ED visits, but may have merit when predicting IH visits especially for patients with an insomnia diagnosis. The results also indicate that using both diagnosis and medications as a source of data does not generally improve model performance and may increase its overhead. Based on these findings, the proposed evaluation methodologies are recommended to ascertain the utility of disease-specific models in addition to the traditional intra-cohort testing.

## Introduction

Interest in machine learning (ML) has significantly increased in the health sector over the past decade. The fields of neurology, radiology, ophthalmology, anesthesia, intensive care medicine and oncology have all explored ML^1^. While these ML models show potential in experimental settings, their use in real-world practice is limited. To successfully translate ML models from research to practice, the constraints and limitations of the models in a production environment must be fully understood.

Most ML models in healthcare focus on two main outcomes: chronic disease risk prediction^2^ and acute care utilization^3^. The sources of the data for these models vary considerably as does the focus on either diseases or procedures. Data modalities ranging from gene expression, signal data from mobile devices, to laboratory and electronic health record (EHR) data have been used in previous studies^4,5^. Disease-specific random forest (RF) and convolutional neural network (CNN) models have successfully been developed to detect sleep disorder in asthma patients and achieved accuracies of 0.81 and 0.95, respectively^6^. Procedure-specific models tend to focus on post-operative risks. For example, a logistic regression (LR) model was used to predict 90 days readmission after total joint arthroplasty with an area under the receiver operating curve (AUC) of 0.65^7^.

Disease-specific models are common, but often not practical. Using training and validation data from a highly specific cohort of patients may improve model accuracy but it may also reduce generalization to different populations^5^. Because disease-specific data is limited, especially if it is sourced from a single health care institution, models developed using these small datasets are prone to overfitting, making them unsuitable for unobserved patient populations^4^. Moreover, when considering the deployment of the models in practice, use of disease-specific models over general ones may require health care institutions to deploy multiple models, one for each disease of interest. On the one hand, the large number of chronic diseases and maintenance of associated models is a high burden for the health care institutions. On the other hand, selectively choosing among the available ML models goes against the intended ubiquity promise of digital health.

Developing general patient models to predict the risk for multiple disease conditions is difficult. However, a general patient model that can predict acute care utilization (ACU) may be possible if a pragmatic design methodology is followed. ACU is defined as the use of hospital services in the form of emergency department (ED) or inpatient hospital (IH) visits. In this study, we developed insomnia-specific and general patient models to predict ED and IH visits using data from multiple healthcare institutions. The models are then compared using a structured methodology.

## Background

Managing ACU is important for both health care institutions and patients. For health care institutions, the concern is operational efficiency and the aim is to manage surges in demand by increasing resources, reducing the length of stay or redirecting patients. Few operational support models have been proposed to predict hospitalizations, and most are specific to individual healthcare institutions. For example, a neural network model was developed to predict surges in demand for ED services by patients with chronic pulmonary diseases^8^. This short-term forecast model relies on weather and environmental conditions specific to the geo-location of the health care institution. A long-term forecast model for ED visits by patients with non-communicable diseases was also proposed^9^. This model is based on two assumptions: (a) non-communicable diseases are the most important reason for ED visits and (b) the incidence of this type of disease increases with age. Other models focus on predicting the transition from ED to IH visits^10,11^. Although these transition models include patients demographics and medical history as exposure variables, they rely heavily on non-generalizable social determinants (e.g., zip code, mode of arrival) thereby making the model less transferable to other communities^10^.

Identifying the determinants of high ACU for each individual patient was the focus of numerous previous studies. A systematic review of statistical models of ED visits by older adults found that measures of need such as prior ED or IH visits are statistically significant determinants of future ED visits^12^. Other LR models for ACU in the general patient population used exposure variables defined in the adjusted clinical group (AGC) scoring instrument^13^. These variables include demographics, utilization, diagnosis and medication variables with values derived from claims data.

Institution- and patient-centric models for ACU are complementary and serve different purposes. The institution-centric model is unlikely to transfer to other health care institutions. The patient-centric model, if designed properly, may transfer to patients in other health care institutions. Both institution- and patient-centric models can be designed for the general patient population or a subset of this population (e.g., specific disease condition or age group). However, the more specific the target population, the higher is the deployment burden on the health care institution.

The ability to understand, estimate or potentially reduce ED and IH visits in any patient population can have a significant impact on the health care system. Differences in cost between inpatient and outpatient health services are easily measurable and provide a strong supporting argument for adopting ACU models into clinical practice. Moreover, early identification of the small percentage of patients that are high acute care users is critical because they account for a large proportion of the health care costs^14^. However, the proposed models must be scalable and preferably applicable to the wider patient population. If unnecessary, targeting a specific disease condition or age group may perpetuate some of the practices specific to survey-based instruments, introduce unneeded operational burden and infringe on the added benefit afforded by new modeling techniques.

One of the few earlier studies that compares general patient ML models to disease-specific models aims to predict hospital readmission within 30 days^3^. This outcome is different from the two outcomes considered in the present study. However, this earlier study shares some of the practical deployment concerns with the present study; concerns which are often ignored by other ML studies.

The above-mentioned readmission study collected 3.3 million hospital admissions from the New Zealand hospital system over a 6 year period starting from 2006. These data were used to develop models from a multi-modal feature space that included:

- Socio-demographics: sex, age and race of the patient; number of hospital visits within the past 365 days; and type of health care institution (i.e., public versus private).
- Diagnosis: disease codes are collected for each patient following the International Classification of Diseases-10-AM (ICD-10-AM)^15^, after removal of infrequent codes.
- Procedure: list of procedures associated with each hospital admission are also derived from the ICD-10-AM classification.

A subset of 280 disease groups of interest were identified from a total of 815. The selection criteria was based on high cost/penalty for readmission within the New Zealand health care system. Several ML techniques including LR, RF and support vector machine (SVM) were considered. When all 280 disease groups are modeled collectively, the best AUC (0.82) was achieved by the LR model. When each disease group was considered independently, the AUCs of the best disease-specific models range from 0.57 to 0.95. As a result, the authors concluded that while some disease-specific models may show better performance, they are more likely to suffer from limited sample sizes making the general patient cohort model more practical. In the specific case of five chronic diseases (Chronic obstructive pulmonary disorder, Heart failure, Pneumonia, Acute myocardial infarction, Total hip arthroplasty/total knee arthroplasty), the study was also extended to include a deep neural network (NN) model with three hidden layers. The results indicate that the AUC of these deep NN models was similar to their LR counterparts.

In the present paper, we follow a similar empirical approach for two ACU outcomes: IH and ED visits. We also select an insomnia patient cohort for the purpose of disease-specific modeling. Insomnia is one of the most prevalent sleep disorders, affecting approximately 20–30% of the adult population in the United States^16,17^. It was also shown to increase health care utilization among Medicare beneficiaries^18^. The Diagnostic and Statistical Manual of Mental Disorders, Fifth Edition (DSM-5) defines insomnia as dissatisfaction with sleep quantity or quality that is associated with at least one of the following symptoms: difficulty initiating sleep, difficulty maintaining sleep characterized by frequent awakenings or problems returning to sleep after awakenings, or early-morning awakening with the inability to return to sleep^19^. Insomnia often has a significant negative impact on patient quality of life and has been associated with impaired daytime functioning, increased risk of workplace injury and absenteeism, and fatal injuries^20,21^. In addition to its direct negative impact on health-related quality of life and economic productivity, the selection of insomnia is motivated by the fact that this disease can manifest as a primary medical condition or a common secondary comorbid condition to a wide range of chronic conditions such as depressive or anxiety disorders, Alzheimer Disease and other related dementia, and alcohol and substance use disorders^21–23^. Moreover, pharmacologic therapy for insomnia should be limited in duration and cognitive behavioral therapy is preferred^23^. The complexity of the disease condition and the restricted treatment options are added incentives for the identification of insomnia patients that may become high acute care users.

## Methods

The models developed in the present study are derived from EHR data and use different ML techniques. Routine care EHR was selected as a source of data because it is widely available for all patients. Relying on exposure variables from signal or imaging data (e.g., polysomnography) will limit the number of patients available for the study^1^. More importantly, it will restrict the application of the proposed model to a limited cohort of patients for whom the results of these special diagnostic instruments are available since insomnia diagnosis and follow up do not often require complementary methods, and polysomnography is rarely performed^24^.

All procedures performed in the study were in accordance with the ethical standards of the institutional review board of Indiana University and with the 1964 Helsinki declaration and its later amendments or comparable ethical standards. The experimental protocol was approved by the Institutional Review Board of Indiana University (IRB Number: 11732). The study was exempt from patient informed consent by the Institutional Review Board of Indiana University due to the non-interventional and retrospective nature of the study.

### Study population

The patients in the present study are served by any healthcare system providing data to the Indiana Network for Patient Care (INPC), an operational community-wide electronic medical record. The National Library of Medicine and the Agency for Healthcare Research and Quality have supported the initial development of the INPC. The system currently includes data from 19 hospitals in seven health systems, the Marion County Health Department, regional laboratories, radiology centers, and various physician practices. These hospitals account for over 95% of all beds and ED visits in Indianapolis. The data collected include demographics, laboratory results, emergency department, inpatient, and outpatient encounter data, free-text chief complaint, coded diagnoses and procedures, vital signs, and other data, but not all these data elements are available for every participant. These data are accessible to care providers and researchers through the standardized and centralized Medical Record System of the Regenstrief institute. One of the advantage of the Regenstrief Medical Record System is that patients with multiple medical records from different health care institutions are linked. For instance, when a patient is seen in any of the 19 emergency departments operated by the consortium of hospitals, the information from all of these health care institutions about one patient can be presented as one virtual medical record.

Two different cohorts are considered: Insomnia patients and general patients. All patients in the two cohorts must be at least 18 years of age and have at least one encounter on record in the Medical Record System every year from 2010 to 2019. This ensures that selected patients are adults, enrolled in the network and are using the health services over the study period. The insomnia patients are identified according to two criteria:

- A new insomnia diagnosis, or
- A new insomnia medication order.

Insomnia diagnosis is established using a list of ICD^25^ codes in the patient records. Similarly, a list of FDA and non-FDA approved insomnia medications was collected from the literature and used to identify patients that have medication orders consistent with the target list. This list includes zolpidem, suvorexant, butabarbital, quazepam, estazolam, flurazepam, triazolam, tasimelteon, eszopiclone, temazepam, ramelteon, secobarbital, zaleplon, chloral hydrate, and melatonin. Extending the medication list to both FDA and non-FDA approved medications allows the inclusion of undetected insomnia patients in the study cohort. The index date for each insomnia patient is defined as the date of the earliest one of the above two selection criteria. Only patients with index date between January 1st, 2011 and December 31st, 2019 (the study period) were considered in order to ensure that each patient had at least one year of history data prior to the index date.

The study focuses on understanding ACU for incident cases of insomnia. Moreover, antidepressants (e.g., doxepin, trazodone, and mirtazapine) and low-dose antipsychotics (e.g., quetiapine and olanzapine) with hypnotic properties were excluded from the list of medications used for insomnia diagnosis in the present study. These off-label treatments may not be specific to insomnia as they are also prescribed for depression, pain, psychosis and other medical conditions. Verification of off-label medication use for insomnia treatment requires a review of the medical notes. Extending the study to ACU for prevalent cases of insomnia and off-label medications is being considered as part of our future work.

The second cohort consists of general patients that do not satisfy any of the above two criteria over the study period. For each insomnia patient, a matching general patient was selected from the Medical Record System. The match was performed based on age at index date (within one year), sex and race. When multiple matches are available, one patient is selected at random.

For notation simplicity, the insomnia patients are labeled “cases” and the general patients are labeled “controls” in the remainder of the present paper. Moreover, the entire cohort of either cases or controls is labeled “all”, the subset of cases with an insomnia diagnosis (i.e., excluding cases that have been prescribed insomnia medications with no documented insomnia diagnosis in their EHR) is labeled “ICD” and the subset of patients 65 and older is labeled (65+).

### Exposure variables

Exposure variables from routine EHR data are grouped according to three modalities:

- Socio-demographic: Variables in this modality include age, sex, race, ethnicity, insurance type (i.e., government, commercial, self-pay, and other/unknown) and the neighborhood deprivation index^26^ which is derived from the last patient’s address on file at the end of the study period (2019). Three variables are added to this list: the number of ED and IH visits during the year prior to the index date and the Elixhauser’s comorbidity score^7^. This score corresponds to the weighted sum of the 31 Elixhauser’s comorbidity categories^27^. It is widely used for mortality prediction associated with various comorbid conditions (e.g., cancer^28^, dementia^29^ and congestive heart failure^30^).
- Diagnosis: The disease conditions in the patient’s medical record are collected over a period of one year prior to the index date (non inclusive). The disease ICD codes are then aggregated and mapped to the 31 Elixhauser’s comorbidity categories which range from congestive heart failure to depression. This specific comorbidity index was selected because it is based on a widely used disease code taxonomy and includes disease categories related to mental health. Alternative comorbidity indices were considered. Both the Charlson Comorbidity Index^31^ and the NCI Comorbidity Index^32^ have limited emphasis on mental health diseases and the latter was customized for cancer patients.
- Medications: A similar data aggregation approach to that used for the diagnosis modality is applied to the medication orders. For each patient, the medications in the patient’s record during the year prior to the index date are mapped to the appropriate Anatomical Therapeutic Chemical (ATC)^33^ first level subgroup. There are fourteen main groups under the ATC taxonomy. Each group corresponds to a biological system (e.g., nervous system or respiratory system) and may have from 3 to 16 subgroups. For example, the nervous system group has seven subgroups including anesthetics and analgesics. A total of 86 medication exposure variables are developed where each variable corresponds to an ATC medication subgroup. The mapping was performed by using the RxNav API^34^. Some of the ATC subgroups were omitted because they are not relevant to this study (e.g., diagnostic agents, contrast media, diagnostic radiopharmaceuticals, etc.). Although other medication taxonmies are available (e.g., the Generic Product Identifier, the USP Drug Classification and the First Databank’s Drug Classification), the choice of the ATC taxonomy was motivated by its wide-use and hierarchical structure. The first characteristic was necessary to support the design of a generalizable model for patients from multiple health care institutions and the second was needed to facilitate the aggregation of different types of medications.

### Predictive models

The exposure variables described in the previous section are used to predict the ACU for cases and controls during the year following the index date. Since the control patients are selected randomly, this cohort is representative of the general population of patients affiliated with all the health care institutions contributing data to INPC. Moreover, matching between cases and controls was based on age, sex and race. Therefore, potential biases inherent to the insomnia population due to these variables are reduced. Two outcomes are analyzed: ED and IH visits. Each outcome is stratified into two classes: (1) zero visit and (2) one or more visits. This stratification corresponds to a binary classification. A regression model where the outcome is the number of visits was also considered. However, given the low number of patients with one or more visits compared to the number of patients with zero visits in the study cohorts, binary classification was best suited for identifying at risk patients of ACU. Using this binary stratification, different models were developed by varying the cohort, the exposure variables and the outcome variables.

In a first step the socio-demographics variables are used to train models using cases and controls independently. These models are compared to models that are developed using the combinations of (a) the socio-demographic and the diagnosis variables and (b) the socio-demographic and the medications variables. The purpose of this comparison is to identify differences in entropy among the socio-demographics, diagnosis and medication modalities. A global model that combines all the exposure variables is also developed. Again, the purpose of this step is to identify the modality and the variables with the highest predictive power for ACU in insomnia and control patients.

In previous studies, the prevalence of insomnia was found to vary depending on age^23^. Moreover, previous research suggest that more disease-specific cohorts tend to be more homogeneous^3^. In order to validate these findings through sensitivity analysis, additional models are developed for two sub-cohorts: 65+ years patients and ICD cases.

For all scenarios, the models induced by the insomnia cases are compared to the models induced by the control patients. The goal of this comparison is to establish whether or not an insomnia-specific model for ACU is necessary. This assessment is validated using a technique inspired by transfer learning^35^. In brief, transfer learning is a process that aims at transferring knowledge from one domain to a related domain. For instance, transfer learning was used to fine tune an image classification model for dementia^36^. Since a limited number of MRI image samples are available for dementia patients, the authors started with a pre-trained model for classification of general purpose images. They subsequently fine-tuned this model to the specific task of identifying MRI images for dementia patients using a limited number of samples. In the present paper, the idea of transfer from one domain to another is used for the purposes of validation and testing. Transfer testing is used to evaluate the performance of models trained using a general patient cohort when tested on a cohort of insomnia patients. If the observed performance is equivalent to that of a model natively trained on insomnia patients, then one can stipulate that an insomia-specific ACU model is not needed.

For all of the above experiments five different ML techniques are investigated: SVM, RF, Neural Networks (NN), LR and Extreme Gradient Boost (XGB) in order to assess the findings with respect to a wide range of ML techniques. Moreover, all models are evaluated using a single metric: AUC. This metric was selected because it is a measure of the ability of the models to discriminate between the two outcome classes independent of any threshold value. The architecture and hyper-parameters of the models are included in Table 1. For each model, the hyper-parameters were fined-tuned using a cross-validated grid search over the training dataset with AUC as the optimization metric.

**Table 1:** Hyper-parameters and architecture of the models.

Model	Hyper-parameters
SVM	Kernel = RBF, C = [0.1–10], Gamma = [0.001–5]
RF	Number of estimators = [50–300], Maximum depth = [5–30], Minimum samples split = [2–15], Minimum samples leaf = [1–15]
NN	Number of layers = [1–5], Number of nodes per layer = [10–100], learning rate = [0.01–0.05], Optimizer = Adam
LR	L2 penalty with C=1.0
XGB	Number of estimators = [30–200], Maximum depth = [3–5], Minimum child weight = [1–10], Gamma = [0.5–5], Learning rate = [0.01–0.05]

Optimal hyper-parameters values were obtained using a grid search over cross-validated models for each architecture.

## Results

The selection criteria described in the previous section identified 18,814 insomnia cases (Table 2). These were matched with 18,814 controls. Of the total number of cases, 65% had no ED visits (Class 0) during the year after the index date and the remaining 35% had one or more ED visits (Class 1). Similarly, of the total number of controls, 78% had no ED visits and the remaining 22% had one or more ED visits. This class imbalance was also observed for the IH visits as shown Table 2. Adequate training of ML models is best achieved with balanced datasets. Moreover, to support a rigorous comparative analysis between disease-specific and general patient models, the number of patient records used to develop and test the models had to be consistent. As such, the development and testing of the ED and IH models included 4190 and 2523 patients from each class for a total of 8380 and 5046, respectively.

**Table 2:** Number of patients in Class 0 (0 visits) and Class 1 (one or more visit) for the insomnia patient cohort (cases) and the general population cohort (controls).

	Cases	Controls
ED visits		
Class 0	12,130 (65%)	14,624 (78%)
Class 1	6684 (35%)	4190 (22%)
IH visits		
Class 0	13,803 (73%)	16,291 (87%)
Class 1	5011 (27%)	2523 (%13)

Typically for ML models, 80% of the data is used for training while 20% is used for testing. In this study, because of the attrition due to the sensitivity analysis for patients 65+ years old and the insomnia cases with ICD diagnosis code, a 70/30 split was adopted to allow for sufficient test samples. The split in number of patients for the age-specific models (65+) and the (ICD) cases models are obtained by applying the appropriate selection criteria to the (all) cases or controls datasets. The number of development and test patients for all models are shown in Table 3.

**Table 3:** Number of patients in training and testing datasets for each model.

	Cases (all)	Controls (all)	Cases (65+)	Controls (65+)	(ICD) cases
ED visits					
Train	5866	5866	2200	2200	3407
Test	2514	2514	944	944	1461
IH visits					
Train	3532	3532	1615	1615	2633
Test	1514	1514	693	693	1129

Table 4 includes the AUC of the models that use only the sociodemographic (So) variables. The AUCs and the 95% confidence interval using bootstrapping are obtained over the test datasets. The top section of the table is dedicated to ED visits while the bottom section reports the classification results for IH visits. The AUC for the ED cases models varies between 0.56 and 0.60 compared to a range between 0.67 and 0.71 for the ED control models. A slight drop in performance is observed when the patient population is limited to the age group 65+ for both cases and controls. This drop may be due to the difference in number of samples used in the (all) and the (65+) models. Table 4 also shows that the AUC of the models induced by the (all) cases and (ICD) cases cohorts are comparable despite the lower number of samples used to develop the latter models. Moreover, the results in Table 4 indicate that the (all) cases and controls models have a confidence interval (CI) with a range less than 6% whereas for the 65+ models, the CI range is greater than 6% indicating that the (all) models have a higher level of certainty. Similar trends are observed for the IH visits.

**Table 4:** Intra-cohort evaluation: AUC and 95% confidence interval of test samples for models trained on sociodemographic variables (So).

	Cases (all)	Controls (all)	Cases (65+)	Controls (65+)	Cases (ICD)
ED visits					
SVM	0.60 [0.58, 0.63]	0.69 [0.67, 0.71]	0.56 [0.53, 0.60]	0.65 [0.62, 0.69]	0.57 [0.54, 0.60]
RF	0.60 [0.58, 0.62]	0.71 [0.68, 0.71]	0.59 [0.55, 0.62]	0.66 [0.63, 0.70]	0.58 [0.56, 0.61]
NN	0.57 [0.55, 0.59]	0.69 [0.67, 0.71]	0.57 [0.54, 0.61]	0.64 [0.61, 0.67]	0.56 [0.54, 0.60]
LR	0.56 [0.54, 0.58]	0.67 [0.65, 0.69]	0.58 [0.55, 0.62]	0.66 [0.63, 0.70]	0.56 [0.53, 0.58]
XGB	0.60 [0.58, 0.63]	0.71 [0.69, 0.73]	0.59 [0.55, 0.63]	0.67 [0.64, 0.70]	0.60 [0.57, 0.62]
IH visits					
SVM	0.62 [0.59, 0.65]	0.65 [0.62, 0.68]	0.55 [0.50, 0.59]	0.63 [0.58, 0.67]	0.63 [0.59, 0.66]
RF	0.66 [0.64, 0.69]	0.69 [0.66, 0.71]	0.60 [0.56, 0.64]	0.66 [0.61, 0.70]	0.68 [0.65, 0.71]
NN	0.59 [0.56, 0.62]	0.65 [0.62, 0.68]	0.57 [0.53, 0.61]	0.62 [0.58, 0.65]	0.61 [0.58, 0.64]
LR	0.60 [0.57, 0.63]	0.63 [0.60, 0.66]	0.57 [0.53, 0.61]	0.64 [0.60, 0.68]	0.64 [0.62, 0.68]
XGB	0.66 [0.64, 0.68]	0.69 [0.66, 0.71]	0.57 [0.53, 0.61]	0.65 [0.61, 0.69]	0.69 [0.66, 0.73]

The AUC of the models that use the disease conditions variables (Dx) in addition to the So variables are reported in Table 5. When compared to the So-only counter-part models (Table 4), the results show an improvement in AUC for all models. The increase in AUC is 0.10 or higher for most models. The cases (ICD) models benefited from the highest improvement with an increase of more than 0.15 in AUC. This increase in performance is attributed to the added entropy from the disease conditions modality. The CI range of the models after the addition of the Dx modality is consistent with the baseline So model. Moreover, the ED cases models (columns 1 and 3) still have a lower performance than the ED controls models (columns 2 and 4). For the IH visits, with the addition of the Dx variables, the cases models show higher AUCs than the corresponding controls models. The opposite was true for the models that only used the So variables. The best IH models are the (ICD) cases models with AUCs ranging between 0.80 and 0.95 for all ML techniques.

**Table 5:** Intra-cohort evaluation: AUC and 95% confidence interval of test samples for models trained on sociodemographic variables (So) and Elixhauser comorbidity conditions (Dx).

	Cases (all)	Controls (all)	Cases (65+)	Controls (65+)	Cases (ICD)
ED visits					
SVM	0.70 [0.67, 0.71]	0.75 [0.73, 0.77]	0.69 [0.65, 0.72]	0.74 [0.71, 0.77]	0.69 [0.66, 0.71]
RF	0.69 [0.66, 0.71]	0.79 [0.77, 0.81]	0.69 [0.65, 0.73]	0.75 [0.72, 0.79]	0.73 [0.71, 0.75]
NN	0.69 [0.67, 0.71]	0.79 [0.77, 0.80]	0.66 [0.63, 0.70]	0.75 [0.73, 0.79]	0.72 [0.70, 0.75]
LR	0.68 [0.66, 0.70]	0.77 [0.76, 0.79]	0.68 [0.64, 0.71]	0.75 [0.72, 0.78]	0.71 [0.68, 0.74]
XGB	0.70 [0.68, 0.72]	0.79 [0.77, 0.81]	0.69 [0.66, 0.73]	0.75 [0.72, 0.79]	0.73 [0.70, 0.75]
IH visits					
SVM	0.76 [0.74, 0.78]	0.76 [0.73, 0.78]	0.73 [0.69, 0.77]	0.73 [0.69, 0.77]	0.80 [0.77, 0.82]
RF	0.80 [0.77, 0.81]	0.78 [0.76, 0.81]	0.75 [0.71, 0.79]	0.76 [0.73, 0.80]	0.95 [0.94, 0.97]
NN	0.78 [0.76, 0.80]	0.77 [0.74, 0.80]	0.74 [0.70, 0.78]	0.75 [0.71, 0.79]	0.82 [0.79, 0.85]
LR	0.76 [0.73, 0.78]	0.74 [0.72, 0.77]	0.73 [0.69, 0.77]	0.74 [0.70, 0.78]	0.82 [0.80, 0.84]
XGB	0.80 [0.78, 0.82]	0.79 [0.77, 0.82]	0.74 [0.70, 0.78]	0.76 [0.72, 0.80]	0.95 [0.94, 0.97]

The performance of the models that combine the So variables with the ATC medication groups variables (Rx) is shown in Table 6. Compared to the baseline So models (Table 4), the addition of the Rx variables leads to an increase in performance similar to that observed after the addition of the Dx variables. Moreover, most of the trends observed with the addition of the Dx variables are maintained when the So variables are combined with the Rx variables. The predictive performance of the models in Table 5 is also equivalent to that of the corresponding models in Table 6. The exception is the (ICD) cases models based on RF and XGB for IH visits where the improvement in AUC afforded by the addition of the Dx variables to the baseline model is 0.25 compared to 0.15 with the addition of the Rx variables. The first finding is interesting but not surprising since medications and disease conditions are clinically related. However, the practical implication of this result is significant because it indicates that one of these two modalities is sufficient. To confirm this finding, models that use all three modalities are developed and the corresponding AUC are shown in Table 7. The fact that the Dx variables have better predictive performance than Rx variables for future IH visits by (ICD) cases suggests that patients with documented insomnia may share similar comorbid conditions which are important for IH visits.

**Table 6:** Intra-cohort evaluation: AUC and 95% confidence interval of test samples for models trained on sociodemographic variables (So) and ATC medication groups (Rx).

	Cases (all)	Controls (all)	Cases (65+)	Controls (65+)	Cases (ICD)
ED visits					
SVM	0.70 [0.68, 0.72]	0.71 [0.69, 0.74]	0.65 [0.62, 0.69]	0.77 [0.73, 0.79]	0.71 [0.68, 0.73]
RF	0.71 [0.69, 0.73]	0.81 [0.80, 0.83]	0.69 [0.65, 0.72]	0.80 [0.77, 0.82]	0.73 [0.70, 0.75]
NN	0.67 [0.65, 0.70]	0.79 [0.77, 0.80]	0.67 [0.64, 0.71]	0.77 [0.73, 0.79]	0.72 [0.69, 0.75]
LR	0.69 [0.67, 0.71]	0.78 [0.76, 0.80]	0.68 [0.64, 0.71]	0.75 [0.72, 0.78]	0.70 [0.67, 0.73]
XGB	0.71 [0.69, 0.73]	0.82 [0.80, 0.83]	0.68 [0.65, 0.72]	0.79 [0.76, 0.82]	0.73 [0.71, 0.76]
IH visits					
SVM	0.76 [0.74, 0.79]	0.73 [0.71, 0.76]	0.70 [0.66, 0.73]	0.68 [0.64, 0.72]	0.79 [0.76, 0.81]
RF	0.80 [0.77, 0.82]	0.81 [0.78, 0.83]	0.75 [0.72, 0.79]	0.79 [0.75, 0.82]	0.84 [0.81, 0.86]
NN	0.75 [0.72, 0.77]	0.74 [0.72, 0.77]	0.73 [0.69, 0.77]	0.73 [0.68, 0.76]	0.80 [0.78, 0.83]
LR	0.74 [0.71, 0.76]	0.74 [0.71, 0.76]	0.71 [0.67, 0.75]	0.71 [0.67, 0.75]	0.82 [0.80, 0.84]
XGB	0.78 [0.76, 0.80]	0.81 [0.78, 0.83]	0.76 [0.72, 0.80]	0.79 [0.75, 0.82]	0.84 [0.81, 0.86]

The first three columns of Table 7 represent the AUC of the (all) cases and control models and the (ICD) cases models that combine the So, Dx, and Rx variables. The age-specific models are omitted from this experiment because they were found in Tables 5 and 6 to not improve prediction for ED or IH visits. The results in the first three columns of Table 7 confirm that there is no clear benefit from including both the Dx and Rx variables when predicting ED or IH visits. In order to gain further insight into which exposure variables are most predictive when all three modalities are combined, the top 5 features of the (all) cases, (all) controls and (ICD) cases RF models are examined (Table 8). Most of the top variables are So variables. This suggests that while So variables alone have a low entropy (Table 4), they are important when combined with other clinical variables. Moreover, the history of previous ED and IH visits are important in all models. This observation aligns with previously reported findings^3,12^. That said, variables from the medication and disease groups still participate in the classification and some of the medication variables are among the top five (e.g., Analgesics and Psychoanaleptics).

**Table 7:** Intra-cohort and transfer testing evaluation: AUC and 95% confidence interval of test samples for models using sociodemographic (So), Elixhauser comorbidity conditions (Dx) and ATC medication groups (Rx) variables.

	Intra-Cohort			Transfer testing	
	Cases (all)	Controls (all)	Cases (ICD)	Cases (all)	Cases (ICD)
ED visits					
SVM	0.70 [0.68, 0.72]	0.73 [0.72, 0.75]	0.72 [0.70, 0.74]	0.61 [0.59, 0.63]	0.70 [0.67, 0.72]
RF	0.71 [0.69, 0.73]	0.81 [0.80, 0.83]	0.74 [0.71, 0.76]	0.67 [0.65, 0.69]	0.70 [0.68, 0.73]
NN	0.68 [0.66, 0.70]	0.79 [0.77, 0.80]	0.72 [0.69, 0.74]	0.66 [0.65, 0.69]	0.70 [0.67, 0.72]
LR	0.69 [0.67, 0.71]	0.78 [0.76, 0.80]	0.71 [0.68, 0.74]	0.68 [0.66, 0.70]	0.69 [0.67, 0.72]
XGB	0.70 [0.68, 0.72]	0.80 [0.79, 0.82]	0.73 [0.71, 0.74]	0.70 [0.67, 0.72]	0.71 [0.68, 0.74]
IH visits					
SVM	0.74 [0.72, 0.76]	0.75 [0.72, 0.77]	0.83 [0.81, 0.85]	0.73 [0.71, 0.76]	0.67 [0.63, 0.69]
RF	0.80 [0.78, 0.82]	0.80 [0.78, 0.83]	0.84 [0.82, 0.86]	0.74 [0.72, 0.77]	0.81 [0.79, 0.84]
NN	0.75 [0.72, 0.77]	0.74 [0.71, 0.77]	0.82 [0.80, 0.84]	0.75 [0.73, 0.78]	0.76 [0.74, 0.79]
LR	0.75 [0.72, 0.77]	0.74 [0.71, 0.76]	0.82 [0.80, 0.84]	0.76 [0.74, 0.78]	0.80 [0.77, 0.82]
XGB	0.80 [0.78, 0.82]	0.81 [0.79, 0.83]	0.85 [0.82, 0.87]	0.76 [0.74, 0.78]	0.80 [0.77, 0.83]

**Table 8:** Top 5 variables identified in RF for the Intra-cohort models that use the sociodemographic (So), Elixhauser comorbidity conditions (Dx) and ATC medication groups (Rx) exposure variables.

ED visits	
Cases (all)	History of ED visits, Age, Deprivation index, Analgesics, History of IH visits
Controls (all)	History of ED visits, Age, Deprivation index, Race, Antibactibacterials for systemic use
Cases (ICD)	History of ED visits, Age, Deprivation index, Psychoanaleptics, History of IH visits
IH visits	
Cases (all)	History of IH visits, Age, Deprivation index, History of ED visits, Analgesics
Controls (all)	Age, History of IH visits, Deprivation index, History of ED visits, Insurance type
Cases (ICD)	History of IH visits, Age, History of ED visits, Deprivation index, Fluid and electrolyte disorders

Two important observations related to the structure of the feature space were derived from Tables 5 and 6. First, control models for ED visits have higher predictive power than cases models. Second, cases models for IH visits have better or similar performance than equivalent controls models. These observations can be confirmed using the transfer testing technique discussed in the Methods Section. Basically, the models trained using controls are tested on cases. The last two columns of Table 7 show the result of these tests. They include the AUC for the ED visits prediction when the 2514 (all) and the 1461 (ICD) test cases are submitted to the ED controls models. Similarly, the lower half of the last two columns includes the AUC of the IH visits prediction for the 1514 (all) and the 1129 (ICD) test cases when processed by the IH controls models. To ensure that results are in fact comparable, the test cases used in the transfer testing are the same test patients used in the evaluation of the (all) and (ICD) cases models in Table 7 (intra-cohort testing).

For all ML techniques under consideration, the AUCs of the (all) and (ICD) cases intra-cohort testing are higher or equal to the AUCs of the corresponding transfer testing (i.e., column 1 versus column 4 and column 3 versus column 5). However, the gain in AUC varies depending on the technique and the outcome. For the highest performing technique, XGB, the AUC of the (all) cases ED visits is the same for intra-cohort and transfer testing (column 1 versus column 4) and only a 0.02 difference is observed for the (ICD) cases ED visits. For the same ML technique and IH visits, more than 0.04 drop in AUC is observed between intra-cohort and transfer testings. This indicates that the IH outcome may benefit from a disease-specific model especially for (ICD) cases where a high AUC was obtained with the So and Dx data modalities (Table 5). In general, for each technique and outcome, it is recommended to implement transfer-testing in order to decide if the gain in performance justifies the deployment of a disease specific model.

## Discussion

Access to ML models that are developed from EHR routine care data can help improve health services. However, efficient modeling using EHR data, even for structured data modalities, is difficult because of missing data and inadequate representation of the general patient population in the training and validation datasets^4^. This complexity is compounded by health institution-specific data distributions and patient cohort segmentation along disease conditions or age groups. For ML models to be adopted by health care systems, they must be widely applicable and carry minimum burden. These design considerations are often overlooked. In this study, modeling ACU is used to demonstrate the importance of feature selection, cohort segmentation, and model architecture design decisions when developing ML models for practical use.

First, it is critical to select a limited number of widely available exposure variables. Standard demographic variables include age, sex, race and ethnicity. These variables have limited inconsistencies and are widely available. However, augmenting demographic variables with other social determinants must be done with care. In this study, previous ED and IH visits were included because they were shown to be strong predictors of future need for health services^3^. Social determinants such as insurance or a deprivation index must be appropriately stratified for ease of transfer to other health care institutions. For instance, the stratification of the insurance type in the present study followed government, commercial, self-pay and other/unknown. An alternative insurance stratification is public versus private^3^. The Elixhauser’s commorbidity score is also generalizable since it is calculated using ICD diagnosis codes. Using variables such as zip codes or mode of transportation as social determinants^10^ may limit the generalizability of the model.

In contradiction with previous findings^13^, the present study suggests that the combination of So variables and either Rx or Dx variables is best. Using all three modalities is not necessary. Choosing between Rx and Dx variables can have an impact on the generalizability of the model to other health care institutions. Diagnosis conditions are often documented using ICD codes. Exposure variables derived from ICD codes are likely to be interoperable across health care systems. Moreover, diagnosis conditions are fewer, likely to be persistent and consistent from one health care provider to another. Finally, the diagnosis codes are aggregated by disease groups which further reduces potential for variability.

In contrast, Rx variables may not seamlessly generalize for several reasons. Treatment plans may differ from one health care provider to another even for the same disease condition. Documentation of patients’ medications in the EHR of health care institutions may rely on different classification taxonomies (e.g., GPI, ATC and UPS). Translating from one classification to another is also not trivial and requires substantial manual efforts and guidance from subject matter experts because one-to-one mappings for medication groups in one taxonomy to another taxonomy is not possible. Moreover, the Rx modality evolves as new drugs are introduced and old drugs are discontinued or modified. Therefore, even if a common medication taxonomy is possible, models derived from Rx exposure variables have to be updated on a regular basis. Finally, due to the movement of patients from one health care provider to another, the history of medications compared to the history of diagnosis is more likely to be incomplete in the EHR thereby leading to classification errors. That said, if Rx variables are omitted, some of the acute care utilization directly related to specific medications may be missed.

There are other modalities that have not been considered in the present study. These include medical notes, procedures and laboratory results. Medical notes have been used to predict patients at high risk for ACU^14,37^. However, the transfer of this modality to other patient populations remains an open research question. Laboratory results may not be widely available as in the case of polysomnography data for insomnia patients^1^.

Second, the present study indicates that

- An insomnia-specific model for predicting ED visits is not recommended. In fact, the general patient model is sufficiently accurate for the insomnia test patients.
- Predicting IH visits can benefit from a disease-specific model especially for severe cases of insomnia (i.e., patients that will eventually progress to a documented diagnosis).
- An age-specific model for older adults does not have better performance than the general patient model for predicting either ED or IH visits.

These findings can guide the development of ACU models, general patient models are more practical, cohort segmentation can limit the utility of the models and reduce the data available for their development. Previously, disease-specific prediction models for hospital re-admission were found to outperform general patient models^3^. This finding is consistent with the insomnia-specific model for IH visits presented in this study. However, for ED visits this may not be applicable.

The need for cohort segmentation should be evaluated for every model. As demonstrated in the present study, this can be achieved by comparing the cohort-specific model to an equivalent general patient model. In addition, transfer testing between the two models should be performed to confirm that the predictive performance is improved with segmentation. In the current study, transfer testing was able to confirm that disease-specific segmentation was only beneficial for IH models.

Third, common model architectures used in previous insomnia-related studies include LR, SVM, XGB, RF and NN^1^. All of these techniques were investigated in the present study. Deep NN are generally more complex than others ML architectures. For instance, a stack of denoising auto-encoders was shown to be able to model risks for diabetes, schizophrenia and cancer from EHR data without any feature engineering by human experts^38^. However, other studies found that the performance of NN models was similar to their LR counterparts^1,3,4^. The complexity of deep learning models may not be warranted in practice especially if the exposure variables are well defined. Traditional ML models may be sufficient. Typically, deep learning models are useful when the number of exposure variables is large. Evaluating different architectures is necessary in order to select the most favorable one while taking into account practical considerations such as maintenance and data processing efforts.

## Conclusion

Understanding the deployment constraints of ML models is necessary for their adoption by health care providers. These models can enhance disease diagnosis and enable the systematic classification of disease risks and health care services utilization. However, the design and engineering of these models must take into consideration their added burdens on the already overburdened health care system. This burden can increase with the inclusion of unnecessary and hard to obtain exposure variables and with cohort segmentation. In the present study, these design considerations are illustrated by comparing an insomnia patient cohort to a general patient cohort. The focus of the study is predicting future ED and IH visits for these patients.

Our results indicate that it is not necessary to include both diagnosis and medication history variables when predicting future ED and IH visits. Disease- and age-specific models for predicting future ED visits are also not needed. In contrast, the IH disease-specific models outperform the general patient models. The broader question of whether disease-specific models are necessary remains open. However, this study identified at least a scenario where it is not the case. The utility of any ML model developed for a segment of the population should not be proposed in isolation. We recommend that its predictive performance be compared to the general patient equivalent model. Transfer testing must also be performed to confirm that the cohort-specific model outperforms the general patient model. Our future work will focus on applying the methodologies introduced in the present study to other patient populations including modeling ACU for obstructive sleep apnoea patients as well as predicting patients at risk for obstructive sleep apnoea.

## Data Availability

The data that support the findings of this study are available from the Regenstrief Institute but restrictions apply to the availability of these data, which were used under a research agreement for the current study, and so are not publicly available. Data are however available from the corresponding author upon reasonable request and with permission of the Regenstrief Institute.
